# The Association between Environmental Lead Exposure and High School Educational Outcomes in Four Communities in New South Wales, Australia

**DOI:** 10.3390/ijerph14111395

**Published:** 2017-11-16

**Authors:** Jennifer McCrindle, Donna Green, Marianne Sullivan

**Affiliations:** 1 Climate Change Research Centre, University of New South Wales, Kensington, Sydney 2052, Australia; 2 Climate Change Research Centre and the ARC Centre of Excellence for Climate Systems Science, University of New South Wales, Kensington, Sydney 2052, Australia; donna.green@unsw.edu.au; 3 Department of Public Health, William Paterson University, Wayne, NJ 07470, USA; sullivanm19@wpunj.edu

**Keywords:** soil lead, high school exit outcomes, Australia

## Abstract

The associations between environmental lead exposure and high school educational outcomes in four communities located in New South Wales, Australia, were examined in this ecological study. A mixed model analysis was performed to account for each school’s results being more similar than results for other schools. The effect of environmental lead exposure on mean results for five educational outcomes was examined. ‘Leaded’ schools with more than five per cent of students living in the highest lead risk areas were tested against non-leaded ‘comparison’ schools that were matched by a pre-defined socio-educational advantage rating. A small disadvantage was found for leaded schools for four out of five outcomes, which was statistically significant for three outcomes: Higher School Certificate English (*p* < 0.01), School Certificate Mathematics (*p* < 0.05), and Australian Tertiary Admissions Rank eligibility rate (*p* < 0.01). This study adds to the large body of evidence in Australia and elsewhere supporting the importance of primary prevention to protect health at multiple stages of development.

## 1. Introduction

Lead has long been recognised as a neurotoxin and children are particularly at risk [1,2]. Recent research has proven that even low lead levels can result in significant impairment of neurocognitive functioning [3,4,5]. The effects of lead exposure on educational outcomes and Intelligence Quotient (IQ) have been widely studied [6,7,8,9,10]. There is a causal relationship between low level lead exposure and decreased cognitive function in childhood, demonstrated through studies that show IQ decrements and lowered educational achievement as outcomes of lead exposure [6,7,8,9,10,11].

Many industrialised and urban areas are contaminated with lead as a result of human activities. In Australia, lead was completely removed from petrol in 2002 [12] and paint in 1970 [13], significantly reducing the new lead burden in and around residential areas. In addition to these chronic, diffuse legacy sources of lead, there are several large point sources which are still releasing significant quantities of airborne lead. These sites are either lead smelters (such as Port Pirie and Mount Isa) or lead mines (such as Broken Hill, Mount Isa and Macarthur River).

Australia has national standards for allowable lead concentrations in soils and releases to air [14]. Although these are non-binding for the States and Territories, they are usually used by respective State Environment Protection Authorities (EPAs) to set regulations for permissible concentrations of pollutants. The soil concentration guidelines for lead are documented in the National Environment Protection Measure (NEPM) Assessment of Site Contamination, 1999, which sets a guideline of 300 mg/kg for a standard residential area including primary schools, and 600 mg/kg for developed areas of open space such as parks, playgrounds and secondary schools [15]. It should be noted that these soil lead guidelines were based on a blood lead level of less than 7.5 µg/dL at a time when the National Health and Medical Research Council (NHMRC) recommended a blood lead level of under 10 µg/dL. Despite the NHMRC blood lead level standard being reduced, the NEPM guidelines for soil lead levels have not yet been lowered accordingly.

NHMRC guidelines for allowable blood lead concentrations were updated in 2015, reducing the reference level of blood lead to below 5 µg/dL [16]. The standard method to ascertain lead exposure is to measure blood lead levels. Yet, unlike in the US, in Australia there is no national surveillance system for childhood lead exposure [16] and the frequency of blood lead level assessments conducted in mining or smelting communities varies. The pathway of human lead exposure from soil contamination is well established [17,18] and soil lead levels are a reliable proxy for lead exposure as there is a strong association between soil lead contamination and blood lead levels [19,20,21]. The NHMRC supports this, stating that people can be regarded as exposed to lead when there is lead in their “immediate environment (e.g., home or workplace)” [16].

A causal relationship between lead and early childhood cognitive functioning has been proven by numerous studies [6,7,8,9,10,11]. The majority of studies in Australia have focussed on the South Australian town of Port Pirie with extensive studies on health effects of lead exposure, and some research on effects on IQ of children and adolescents [22]. A recent study in Broken Hill found that lead exposure was associated with developmental vulnerabilities in early childhood and lowered National Assessment Program—Literacy and Numeracy (NAPLAN) results [23]. Concerning later educational outcomes such as high school exit outcomes, there has been limited research. There are two studies of educational outcomes at 18 years for birth cohorts who had previously been assessed for dentine lead. Among other effects, one study found an association between lead exposure and lower scores on reading, vocabulary and grammatical reasoning tests [24], and the other found small but significant relationships between lead levels and leaving school without educational qualifications and the mean number of School Certificate subjects passed [25]. A follow-up study of one of these cohorts [26] found that higher childhood blood lead levels were associated with poorer adult cognitive performance.

In this paper, we examined the relationship between lead exposure and high school achievement, and for the first time in Australian research, for high school exit outcomes. There are no known studies of the association between lead exposure and high school exit outcomes in Australia, nor on the effect of lead exposure from non-industrial sources. This study raises awareness of the potential impact of environmental contamination from non-industrial sources on important educational outcomes.

## 2. Materials and Methods

### 2.1. Study Design

An ecological design was used to compare educational outcomes for students attending schools in lead exposed areas to the outcomes of students attending comparison schools in communities with no known exceptional exposures to lead.

### 2.2. Measures

#### 2.2.1. Educational Outcomes

Five educational outcomes with data from the Board of Studies Teaching and Educational Standards (BOSTES) are examined: Higher School Certificate (HSC) English, School Certificate (SC) Literacy-English, SC Mathematics, Australian Tertiary Admissions Ranking (ATAR) and rate of retention to year 12. Australian Curriculum, Assessment and Reporting Authority (ACARA) data was used in selecting comparison schools. HSC English, ATAR eligibility rate and rate of retention to year 12 are high school exit outcomes and were examined using BOSTES data for 2008 to 2014. The two SC subjects, English-Literacy and Mathematics were only examined from 2008 to 2011. A summary of the time frames of the data supplied for the educational outcomes is found in Supplementary Materials Table S1.

The SC was awarded to eligible students after the first four years of secondary education. Six subject areas were tested and data for English-Literacy and Mathematics was used in our analysis. The SC was abolished in 2011, as it did not keep up with changes in education [27]. It is still a useful measure for this study to determine upon which domain lead has a greater effect, mathematics or literacy.

The HSC is the highest educational award in NSW schooling. Students who successfully complete Years 11 and 12 are eligible to gain an HSC [28]. There is no one single result or mark awarded for the HSC and it is not valid to compare results between courses. English is the only compulsory course for all HSC candidates and is the most suitable measure for direct comparison. More than three-quarters of students each year were candidates for English (Standard) and English (Advanced) which are reported on a common scale [28]. The remaining students studied other English courses which are each reported on a separate scale [28].

The rate at which students are retained until the final year of formal school education was also examined. This measure was selected as research indicates that early school leavers are drawn disproportionately from the ranks of low achievers and the number of years in school is a significant predictor of future employment and earnings [29]. Since 2010, NSW students must complete Year 10 (or its equivalent), then remain in approved education or training or a combination of education or training and paid work until the age of 17. The previous allowable school leaving age was 15. Due to complexities associated with this measure, the results should be viewed as preliminary.

ATAR is a measure of a student’s overall academic achievement in relation to that of other students [30]. Student ATAR scores are used by the University Admissions Centre (UAC) to rank applicants for university entrance [30]. To be eligible for an ATAR ranking, a student must satisfactorily complete certain courses. The rate of students who are eligible to receive an ATAR was used as students’ actual ATAR scores are not released for research.

#### 2.2.2. Soil Lead Levels

**Figure 1:**
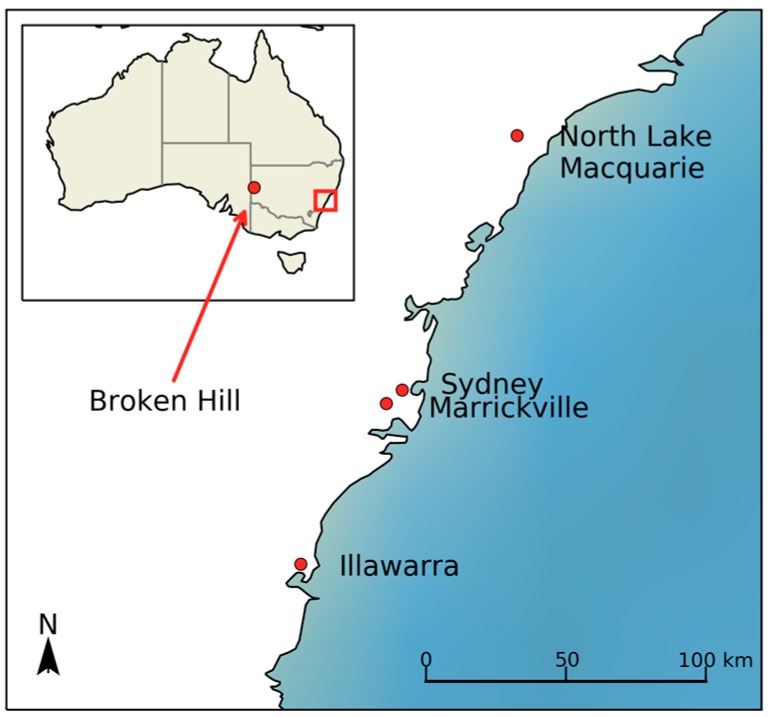
Locator map of four lead exposed New South Wales communities.

The four communities in the state of New South Wales (NSW) that were selected for this study are shown in Figure 1. In this figure, the inset map of Australia shows the location of Broken Hill = in the far west of the state of New South Wales. The main map of part of coastal New South Wales shows the three other lead exposed communities: North Lake Macquarie, Marrickville and Illawarra; as well as the central business district (CBD) of Sydney as a point of reference.

Each of these communities has documented soil lead contamination. The main source of soil contamination in Broken Hill, North Lake Macquarie and Port Kembla includes contamination from an active or recently active smelter or mine [31,32,33]. Secondary sources include paint and leaded fuel [31,32,33]. Marrickville is an inner Sydney suburb where there is a legacy of contaminated soils, mainly from leaded fuel [31].

The environmental exposure of students in this study would have started in early childhood in the early 1990s. In the three areas with industrial sources of lead, the exposure would have been higher than current exposure due to subsequent remediation and abatement, as well as the discontinuation of smelting in North Lake Macquarie and Port Kembla. As well as any early childhood exposure, there would also be concurrent exposure, that is lead exposure occurring at the time of the educational measures which has a strong association with IQ in the primary school years [4].

Previously published soil lead studies were used to demonstrate soil contamination and are summarised in Table 1. The data, where possible, demonstrates soil lead levels during the early childhood years of the cohort and concurrent, or close to concurrent, soil lead levels. In Table 1, we show the bounds of the contaminated areas and the highest lead risk areas (HLRAs).

In Broken Hill, several studies through the 1990s to early 2000s measured lead levels in the soil, air and dust city measuring soil lead levels and identifying lead risk zones. In North Lake Macquarie, the three suburbs in closest proximity to the former smelter in Cockle Creek are highly contaminated [36,37,38] even after an abatement program [37]. Although some surrounding areas were named at risk [43], there is little soil lead data beyond the three closest suburbs. In the Port Kembla area, the soil contamination is highest within 1 km of the former smelter, with a significant soil lead contamination to 4 km from the stack [39,40]. One study noted that the contamination rapidly decreased within the first kilometre, and then the decline was more gradual [40].

In contrast to the three previous regions with an industrial source of lead, Marrickville is one of many inner Sydney suburbs that have a legacy of soil lead contamination from a history of urbanisation, heavy traffic and lead in paint used in older housing. Numerous studies through the 1990s and early 2000s consistently found inner Sydney soil lead levels above 300 mg/kg, with between 33 per cent and 68 per cent of samples in each study [31]. A 1995 study of different categories of residential properties found that about 40 per cent of soil samples from old areas near busy roads, such as Marrickville, exceed 300 mg/kg [41] and more recently the mean soil lead in Marrickville itself was 689 mg/kg [42].

#### 2.2.3. Classification of the Highest Lead Risk Areas

The measured soil lead concentration, used as a proxy for lead exposure, determined the selection of schools from which to examine educational outcomes. Department of Education (DOE) data, BOSTES data and Australian Bureau of Statistics (ABS) data was used to determine the percentage of students living in highest lead risk areas (HLRAs). The ABS’s smallest unit of area for data output is the Statistical Areas Level 1 (SA1). The SA1s were identified (listed in Supplementary Materials Table S2) for the areas of highest lead contamination as described in Table 1. The SA1s were supplied to DOE and BOSTES who then provided available data on student numbers for these highly contaminated areas, which is presented in Table 2.

Table 2 shows a summary of students in the leaded schools and those living in HLRAs. Data from two sources is shown as neither source alone is a complete picture of lead exposure of students at the leaded schools. Each source measured student numbers differently and there is a notable discrepancy between the two sources. The second and third columns show DOE government school data on enrolled year 12 students in each leaded school in each region. The fourth and fifth columns show BOSTES data on students who obtained a result for HSC English. The BOSTES numbers are lower as not all enrolled students would have obtained a HSC English result. BOSTES also provided data on student numbers for the four other educational outcomes (see Supplementary Materials Table S3). HSC English data is shown here, representative of all the educational outcomes.

For the percentage of students in HLRAs, the DOE provided data by school (found in Supplementary Materials Table S4) which was matched to the high lead risk suburbs for each school. On the other hand, BOSTES provided data by SA1s for each community. This data included a small number of HLR students from schools that were eliminated from our study. The percentage of students from eliminated schools was calculated and subtracted, leaving an estimate of the percentage of HLR students for all the educational outcomes (See Supplementary Materials Table S4 for data and calculations).

Despite the proximity of a school to a source of lead, the number of students from the SA1s of highest lead contamination can be very low, in some cases less than one per cent. Therefore, only schools where an average of more than five per cent of enrolled Year 12 students were living in the highest lead SA1s were selected for this study. The five per cent cut off was selected as higher cut off would have greatly reduced the sample size, and a lower cut off would have diluted the effect of lead. Non-government schools, for which there is no DOE data on the numbers of students living in highest lead SA1s, are excluded.

NSW public schools have a defined enrolment area, although students may enrol from out-of-area [44]. DOE provided the in-area enrolment rates (see Supplementary Materials Table S4). For most schools studied, the in-area enrolment remains at or above the state level of 60 to 70 per cent, indicating that including the students in the highest lead SA1s, a high proportion of students are likely to have had some lead exposure. The exception is Marrickville High School which has a considerably lower in-area enrolment. Out-of-area Marrickville students, if living in surrounding parts of inner Sydney, should be assumed to be lead exposed as surrounding inner city LGAs are also lead contaminated [31,41,42].

#### 2.2.4. Classification of “Leaded” and “Comparison” Schools

“Leaded schools” are defined as those which had five per cent or greater of enrolled Year 12 students living in the highest lead SA1s, and 60 to 70 per cent of the school population are assumed to have some lower level of lead exposure. There were seven schools, seen in Table 2, which met these criteria.

To account for the potential influence of socio-economic factors on achievement, the ACARA generated Index of Community Socio-Educational Advantage (ICSEA) was used to identify groups of “comparison schools” with which to compare the leaded schools. The ICSEA value, a rating of socio-educational advantage, measures key factors from data from the families of students enrolled in the school which correlate with educational outcomes. Variables include parental income and education, geographic location of school and proportion of indigenous students [45]. The ICSEA measure allows for direct comparisons to be made between schools that are matched according to their socio-educational advantage [45].

ACARA identifies comparison school groups (CSGs) for every school in Australia by matching a school’s ICSEA value to a group of up to 60 schools containing students from statistically similar backgrounds [44]. We accessed the CSG list for each of the leaded schools then removed primary schools and schools from other states, leaving between two and seventeen statistically similar high schools in NSW for each of the schools in this study (listed in Supplementary Materials Table S5). Schools are matched annually as a school's ICSEA rating may change each year.

The approach of using the ICSEA rating helps to overcome a lack of data for confounding factors, a noted limitation of ecological studies such as this. This approach presents other limitations including the short time span for which comparison school data is available and the low number of schools in the comparison school groups some years. These two factors which reduce the sample size do not appear to impact the results, but may reduce the reliability of this study.

Data are not available to determine individual students’ actual lead exposure through blood lead level testing. Nor is it possible to access information on place of residence in early childhood to determine the likelihood of early childhood lead exposure. Dating back to birth years of the cohorts studied, the ABS has limited data on internal migration, at Statistical District (SD) level, which was the largest geographic unit. Although slightly more accurate data at Statistical Area 2 (SA2) level is available for the past ten years, these SA2s do not correspond with the schools’ enrolment areas, are much larger geographic areas than the schools’ enrolment area, and some enrolment areas are split between two SA2s. As suitable data are not available on the mobility of students, our assumption is that enrolled Year 12 students would have lived in the same residence since birth and would have had early childhood as well as concurrent lead exposure. It is also assumed that comparison school students would have had no exceptional exposures to lead and that any movement of students, whether leaded or comparison school students, would be random.

#### 2.2.5. Statistical Analysis

The statistical analysis was performed using analysis of variance (ANOVA) of two linear mixed models using the lme4 package [46] and R version 3.2.2. The null hypothesis is that the mean scores, weighted by sample size, for leaded schools are not significantly different from comparison schools. First, the full linear mixed model was fit by maximum likelihood. The school was modelled as one random effect. A second random effect was modelled to account for the non-independence of a school’s mean scores each year. Then a second model was fitted with the school type (leaded or comparison) as the fixed effect. The linear mixed models included a random intercept and ANOVA was used to compare these two models with a likelihood ratio test. Significance levels were set at α = 0.05.

#### 2.2.6. Ethics Approval

As part of the requirements of the National Statement on Ethical Conduct in Human Research (Section 5.5.5), this project was approved by the University of New South Wales Human Research Advisory Panel: HC15591.

## 3. Results

Our main finding is that for HSC English, SC Mathematics and ATAR eligibility there is a small, but significant, disadvantage for leaded schools. Statistical results for the mixed models are reported in Table 3. This table shows results for leaded and comparison schools, with data weighted for the number of students. The mean result and lower and upper 95% CIs are reported.

There was evidence of a small but significant negative effect of lead exposure on HSC English, SC Mathematics and ATAR eligibility rates (*p* = 0.002, *p* = 0.028 and *p* = 0.001 respectively). The relative size of the effect of lead on each of the educational outcomes was calculated. The mean HSC English result for leaded schools was 0.88 marks, or 2.7 per cent lower in leaded schools than in comparison schools. The mean SC Mathematics result was 0.97 marks, or 1.5 per cent lower in leaded schools than in comparison schools and the mean ATAR of eligibility rate was 6.3 percentage points, or 16.2 per cent lower in leaded schools than in comparison schools.

For SC English-Literacy results, there was a small negative effect of lead exposure (*p* = 0.077) although this was not significant. Even so, the mean SC English-Literacy mark was 0.56 marks, or less than one per cent lower in leaded schools than in comparison schools. In contrast to the other educational outcomes, for the rate of retention to Year 12 there was a small positive effect of lead exposure, although this was not statistically significant (*p* = 0.826). In relative terms, the mean rate of retention to Year 12 was 0.3 percentage points, or 0.5 per cent higher in leaded schools than in comparison schools.

**Figure 2:**
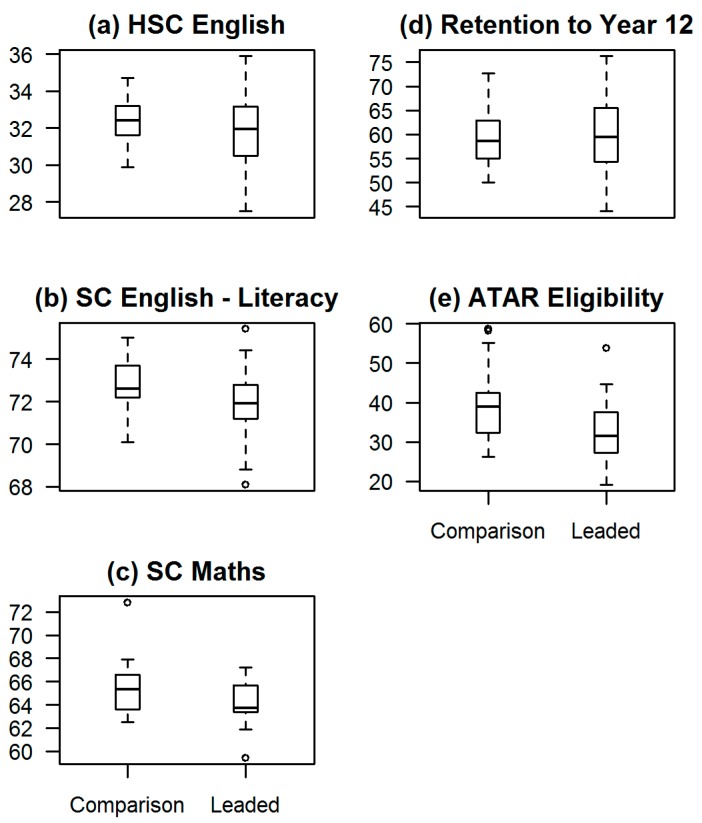
Box plots showing results for the five educational outcomes. (**a**) HSC English; (**b**) SC English-Literacy; (**c**) SC Mathematics; (**d**) Rate of retention to Year 12; (**e**) ATAR eligibility rate.

The results for the five educational outcomes are presented in box plots in Figure 2. For each of the plots, on the *x*-axis is the school type with the category of leaded school on the left and comparison school on the right. The *y*-axis shows the mean for each of the educational outcomes, and it must be noted that the scale for means is not uniform. It is also important to note that SC, ATAR eligibility and retention are measured on a scale to 100, whereas the HSC is measured to 50.

The results for the five educational outcomes are presented in box plots in Figure 2. For each of the plots, on the *x*-axis is the school type with the category of comparison school on the left and leaded school on the right. The *y*-axis shows the mean for each of the educational outcomes, and it must be noted that the scale for means is not uniform. It is also important to note that SC tests, ATAR eligibility and retention are measured on a scale to 100, whereas the HSC is measured to 50.

Unexpectedly, for the three measures of test scores, the range for HSC English and SC English-Literacy is much larger than the range for SC Mathematics. Other than the small sample size, we are not able to ascertain the reason for this.

## 4. Discussion

While a causal relationship [6,7,8,9,10,11] between lead and early childhood cognitive functioning is recognised, there is little research on the association with later educational outcomes such as high school exit outcomes. Our results indicate an association between lead exposure and high school educational outcomes. This is the first study in Australia to indicate that lead exposure may be a risk factor for some aspects of lowered high school achievement.

This study investigated the potential effects of environmental lead exposure on five high school educational outcomes, including exit outcomes of HSC English and rates of ATAR eligibility. There was a small negative effect of lead on four measures, which was statistically significant for three of these measures; the effect being strongest for ATAR eligibility rate and HSC English.

Between the domains of literacy and mathematics, the effect of lead was more pronounced for HSC English. However, lead had a greater effect on SC mathematics than SC English. This mixed result is consistent with previous research, with some studies finding a more pronounced effect for mathematics, and others for literacy. The studies showing a greater effect for mathematics assessed only primary school aged students [8,9,21] including an Australian study in Broken Hill of NAPLAN results [23]. Our study is unique in that it is the first to find a greater effect on mathematics beyond childhood. Interestingly, this contradicts other research in which a greater age range is assessed. One study is of primary aged students [7], one of is for primary and secondary students up to the age of 16 [4] and the other two studies test students at 18 years of age [25,47]. There are also differences in the tests used, with measures such as state [8], national [23] and psychometric [4] tests. Different skills are assessed, for example in the domain of literacy; tests include reading, word recognition, spelling and writing. With differences in ages of the students tested and the type of tests used, this area needs further investigation with a stronger epidemiological study design to be conclusive. Future research could include a review, surveying the specific mathematics and literacy skills tested as well as the ages at which these skills are tested, to better understand the effects of lead on each of these domains at key stages of development.

For the rate of retention to Year 12, there was an unexpected small positive effect of lead exposure, although this was not statistically significant. Due to the complexities of this measure as well as the short time frame, and small sample size, further research is needed particularly as this result contradicts two cohort studies on effects of lead on educational outcomes at 18 years. These studies show, among other effects, a higher risk of dropping out of high school [24], and a relationship between lead and a failure to complete three years of high school [25]. Retention rates need to be investigated as research recommends school dropout become a public health issue [48]. This recommendation is given credence by research including a prospective study showing high school dropouts were 24 times more likely than graduates to experience four or more negative outcomes including poor health and substance abuse [49].

There are several limitations in relation to our design and analysis. First, because this is an ecological study, exposure is measured at the group level based on soil lead levels rather than individual blood lead levels or known lead exposure of individuals. Based on the NHMRC definition of lead exposure, when there is lead in the “immediate environment (e.g., home or workplace)” [16], we assume that students enrolled in schools in areas with significant soil lead contamination are, in fact, lead exposed and that comparison school students are not lead exposed.

Our research has a restricted time frame and sample size due to available data. The results for HSC and SC include only students who completed the tests. Our data, by not including drop outs, is likely to falsely raise leaded schools’ results. Finally, our statistical analysis found significance, but does not go further in assessing the magnitude of the effects of lead exposure on educational outcomes. While our methods of defining leaded schools and assessing the effects of lead are useful, they cannot capture the true extent of the effects of lead exposure. Other than specific cohort studies such as in Port Pirie, in Australia there is currently no capacity to link blood lead levels or even lead exposure risk with health or educational outcome data.

## 5. Conclusions

We found a negative effect of lead exposure on four educational measures, which was statistically significant for three of them, including two high school exit outcomes, consistent with other research that shows that the effects of lead exposure in early life extend beyond childhood. Our results must be interpreted with caution, however, due to the inherent limitations of an ecological design and the analysis limitations discussed above.

Given the impacts of lead on health and educational outcomes, it is imperative that lead exposure in children is prevented. Serious consideration ought to be given to the benefits that would be derived from a coordinated public health approach to childhood lead exposure. This would advance both the generation of and accessibility to data on the extent of the problem and Australia’s progression toward primary prevention [50]. Children should be tested during critical windows of development and exposure to identify those at risk of, or with, elevated blood lead levels. Reducing sources of contamination, preventing exposure through mitigation strategies, and implementation of health and welfare programs, such as Broken Hill’s recently established LeadSmart program, would see likely educational outcomes improve.

## Figures and Tables

**Table 1:** Summary of lead exposure, with the soil lead levels, a brief description of the bounds of the lead exposed areas and the highest lead risk areas.

Region	Summary of Published Soil Lead Levels	Source	Lead Exposed Area	Highest Lead Risk Areas
Broken Hill	Mean soil lead measured in 1992 in the highest lead risk zone 2305 mg/kg	Boreland, 2002 [34]	Urban centre	Two highest of the five lead risk zones
	Mean soil lead in highest lead risk zone 1967 mg/kg	Boreland, 2008 [35]		
	Highest mean soil lead in school catchment area 2865 mg/kg	Dong, 2015 [23]		
North Lake Macquarie	70% of samples in 3 closest suburbs above 300 mg/kg	Galvin, 1993 [36]	Up to 2 km from former smelter 4 km radius from former smelter	Suburbs of Boolaroo, Argenton and Speers Point
	66 percent of soils within 4 km of former stack had soil lead level above 300 mg/kg	Kim, 2009 [37]		
	Post-abatement, in 3 closest suburbs all residential sites except one above 300 mg/kg	Harvey, 2016 [38]	Up to 2 km from former smelter	
Illawarra	highest within 1 km, significant to 4 km significant decrease after 9 km	Martley, 1999 [39]	Suburbs within 9 km of former stack	Suburbs within 4 km of former stack: Port Kembla, Warrawong, Lake Heights, Primbee
	<1 km from site average soil lead 600 mg/kg, beyond 1 km average soil lead 506 mg/kg. Highest levels within 4 km	Jafari, 2009 [40]		
Marrickville	40% of samples in old areas near busy roads in Sydney exceed 300 mg/kg	Olzowy, 1995 [41]	old areas near busy roads	^a^
	Mean soil lead in Marrickville Local Government Area (LGA) of 689 mg/kg	Rouillon, 2017 [42]	Marrickville and some surrounding LGAs	

^a^ No highest lead risk areas (HLRAs) named for Marrickville as the available data was not sufficient to determine the most contaminated Statistical Areas 1s (SA1s). There is no major single point source of lead such as a smelter or mine, and soil lead contamination is likely to be more evenly spread.

**Table 2:** Summary statistics of student numbers in each community, ‘Leaded’ school and in the highest lead risk areas (HLRAs).

Region, School	Number of Enrolled Year 12 Students 2008–2014 ^a^		Number of Students Who Obtained a HSC English Result 2008–2014 ^b^	
	Mean (Range)	Percentage from HLRAs Mean (Range)	Mean (Range)	Percentage from HLRAs (Estimate) ^c^
**Broken Hill**	185 (161–210)			
Broken Hill High School	75 (66–89)	n/a ^e^	61 (40–81)	21.58%
Willyama High School	^d^	n/a ^e^	48 (26–71)	21.58%
**Illawarra**	1147 (1091–1204)			
Warrawong High School	44 (32–48)	35.98% (27.1–44.7%)	26 (14–39)	24.47%
Illawarra Sports High School	118 (89–136)	5.47% (4–12%)	102 (67–131)	24.47%
**North Lake Macquarie**	788 (731–844)			
Lake Macquarie High School	50 (36–59)	14.5% (7.3–22.2%)	36 (25–48)	11.2%
Glendale Technology High School	85 (76–94)	5.69% (1.2–7.1%)	63 (45–80)	11.2%
**Marrickville**	1167 (1029–1286)			
Marrickville High School	42 (33–47)	n/a ^f^	27 (13–34)	n/a ^f^

^a^ source DOE; ^b^ source BOSTES; ^c^ BOSTES data for the percentage of students from HLRAs was provided by region rather than school, thus the leaded schools of a region are combined and have the same percentage. Note that this data is an estimate from the average of all the educational outcomes. ^d^ data not provided; ^e^ missing data for HLRAs as data not available for Broken Hill data provided by suburb name; ^f^ Missing data for the highest lead risk areas in Marrickville as the available data was not sufficient to determine the highest risk Statistical Areas 1s (SA1s). There is no major single point source of lead such as a smelter or mine, and soil lead contamination is likely to be more evenly spread. HSC: Higher School Certificate.

**Table 3:** Summary of the effect of lead on five educational outcomes. Mean, with standard error in parentheses, and 95% confidence intervals for the means.

Educational Outcome	School Type	Mean	Lower 95% Confidence Limits	Upper 95% Confidence Limits	*p* Value
HSC English	Leaded	31.57 (0.272)	30.99	32.14	0.002
	Comparison	32.45 (0.167)	32.12	32.78	
SC Literacy—English	Leaded	72.21 (0.305)	71.52	72.90	0.077
	Comparison	72.77 (0.220)	72.33	73.21	
SC Mathematics	Leaded	64.46 (0.415)	63.40	66.21	0.028
	Comparison	65.43 (0.389)	64.65	65.53	
Rate of retention to Year 12	Leaded	59.68 (1.711)	56.30	63.07	0.826
	Comparison	59.38 (0.758)	57.98	60.79	
ATAR eligibility rate	Leaded	32.65 (1.785)	28.87	36.43	0.001
	Comparison	38.95 (1.082)	36.80	41.11	

SC: School Certificate; ATAR: Australian Tertiary Admissions Ranking.

## Supplementary Materials

The following are available online at www.mdpi.com/1660-4601/14/11/1395/s1, Table S1: Summary of data time frames, Table S2: ABS HLR SA1s-suburb lists, Table S3: BOSTES HLRA student numbers, Table S4: DOE in-area enrolment rates and HLRA student numbers and Table S5: Number of schools in CSGs.
