# Supplementary material for: The Association between Environmental Lead Exposure and High School Educational Outcomes in Four Communities in New South Wales, Australia
Source: Int J Environ Res Public Health. 2017 Nov 16;14(11):1395. doi: 10.3390/ijerph14111395 (PMC5708034; doi:10.3390/ijerph14111395)
Supplement: Supplementary file 1 [file ijerph-14-01395-s001.zip › Table S5 Number of schools in CSGs.pdf]

Number of schools in comparison school groups

School Name	2008	2009	2010	2011	2012	2013	2014	AV
Broken Hill HS	6	3	18	11	7	6	7	8.3
Willyama HS	5	3	13	4	2	12	11	7.1
Lake Macquarie HS	9	8	16	14	16	6	10	9.88
Glendale Technology HS	10	10	19	12	7	9	15	11.7
Warrawong HS	3	4	8	11	17	6	16	9.3
Illawarra Sports HS	8	6	9	7	10	10	11	8.7
Marrickville HS	8	Na #	17	10	5	8	7	9.2
