# Supplementary material for: The Association between Environmental Lead Exposure and High School Educational Outcomes in Four Communities in New South Wales, Australia
Source: Int J Environ Res Public Health. 2017 Nov 16;14(11):1395. doi: 10.3390/ijerph14111395 (PMC5708034; doi:10.3390/ijerph14111395)
Supplement: Supplementary file 1 [file ijerph-14-01395-s001.zip › Table S1 Summary of data time frames.pdf]

Educational Outcome	Years for which data has been supplied	Number of Years
HSC English	2008 -2014	7
Sc English – Literacy	2008-2011	4
SC Mathematics	2008-2011	4
Rate of Retention	2008-2012	5
ATAR Eligibility Rate	2008-2012	5
